# Methylmercury Impact on Adult Neurogenesis: Is the Worst Yet to Come From Recent Brazilian Environmental Disasters?

**DOI:** 10.3389/fnagi.2020.591601

**Published:** 2020-11-23

**Authors:** Ramon da Silva Raposo, Daniel Vieira Pinto, Ricardo Moreira, Ronaldo Pereira Dias, Carlos Alberto Fontes Ribeiro, Reinaldo Barreto Oriá, João Oliveira Malva

**Affiliations:** ^1^Faculty of Medicine, Center for Innovative Biomedicine and Biotechnology (CIBB) and Institute of Pharmacology and Experimental Therapeutics, Coimbra Institute for Clinical and Biomedical Research (iCBR), University of Coimbra, Coimbra, Portugal; ^2^Experimental Biology Core, Health Sciences Center, University of Fortaleza, Fortaleza, Brazil; ^3^Laboratory of Tissue Healing, Ontogeny and Nutrition, Department of Morphology, School of Medicine, Institute of Biomedicine, Federal University of Ceara, Fortaleza, Brazil

**Keywords:** methylmercury, neurotoxicity, neurogenesis, environmental disaster, memory, aging

## Abstract

Worldwide environmental tragedies of anthropogenic origin causing massive release of metals and other pollutants have been increasing considerably. These pollution outbreaks affect the ecosystems and impact human health. Among those tragedies, recent large-scale environmental disasters in Brazil strongly affected riverside populations, leading to high-risk exposure to methylmercury (MeHg). MeHg is highly neurotoxic to the developing brain. This toxicant causes neural stem cell dysfunction and neurodevelopmental abnormalities. However, less is known about the effects of MeHg in the postnatal neurogenic niche, which harbors neural stem cells and their progeny, in the adult brain. Therefore, taking in consideration the impact of MeHg in human health it is urgent to clarify possible associations between exposure to mercury, accelerated cognitive decline, and neurodegenerative diseases. In this perspectives paper, we discuss the neurotoxic mechanisms of MeHg on postnatal neurogenesis and the putative implications associated with accelerated brain aging and early-onset cognitive decline in populations highly exposed to this environmental neurotoxicant.

## Introduction

Methylmercury (MeHg) is considered extremely neurotoxic to the developing brain and chronic exposure to this environmental neurotoxicant may be associated with increased risk of accelerated cognitive decline and neurodegenerative diseases. Indeed, mercury intoxication has been implicated in the etiology of Alzheimer's dementia (Siblerud et al., 2019), putatively causing profound and lasting cognitive decline with aging. Natural and anthropogenic-related environmental disasters may increase the exposure of human populations to mercury intoxication.

Early-life and adult brain neurogenesis in the hippocampus has been claimed to contribute to the cognitive reserve and therefore potentially relevant to better cope with later-life cognitive decline due to physiological aging or under neurodegenerative conditions. In this manuscript, we discuss the possible mechanisms of action of MeHg, a highly toxic organic form of mercury, in the postnatal brain and the underlying implications for accelerated aging and cognitive decline. The fundamental scientific concepts and societal messages should call the attention to environmental disasters, such as the recent large-scale Brazilian environmental outbreaks due to mining activity and its impact on human populations.

### The Environmental Impact of Mercury

Recently there has been a growing concern with the number and impact of environmental disasters worldwide, ranging from volcano eruptions, worrisome forest wildfires, and oil spills. Short- and long-term health consequences to the affected population are still mostly underscored. With aging, the human exposome from accumulating toxicants and biohazards, over the lifespan, may lead to chronic illness, including neurodegenerative diseases (Landrigan et al., 2005; Tshala-Katumbay et al., 2015; Bjørklund et al., 2019).

In Brazil, <5 years ago, two massive mining dams collapsed causing mineral waste dragging into the riverside populations of Mariana and Brumadinho, in the State of Minas Gerais (Almeida et al., 2019; Davila et al., 2020). The latter was especially devastating and considered one of the deadliest disasters of its kind, causing hundreds of deaths and a tremendous environmental calamity. Both disasters led to the exposure of an enormous territory extension, including its fauna and flora, to the mining tailings containing high-level biohazards, such as mercury, as the mineral waste reached the local rivers (Hatje et al., 2017; de Freitas et al., 2019).

Mercury is a ubiquitous heavy metal usually present in low levels in the environment. This compound derives from both natural and anthropogenic sources (Braga et al., 2020). In nature, mercury can be found in inorganic or organic forms, the latter being highly toxic, easily accessible to cells, and able to cross cell membranes, following biomethylation in the form of MeHg (Syversen and Kaur, 2012).

Widespread reducing sulfate bacteria, residing in aquatic sediments, produce MeHg. Moreover, mercury is exploited by artisanal miners to separate gold from other minerals and materials during gold mining (Chételat et al., 2018). This activity, very frequent in the Amazonian region, leads to the accumulation of mercury and release of volatile MeHg to the riverbanks. In the Brazilian Amazonian region a gold rush, since the last quarter of the twentieth century, generated an impressive emission of 120 tons of mercury per year (Berzas Nevado et al., 2010). This super-intense environmental contamination persists nowadays causing a threatening exposure of the Amazonian riverside human populations to this toxic compound (Pinheiro et al., 2008; Berzas Nevado et al., 2010; Arrifano et al., 2018).

**Figure 1:**
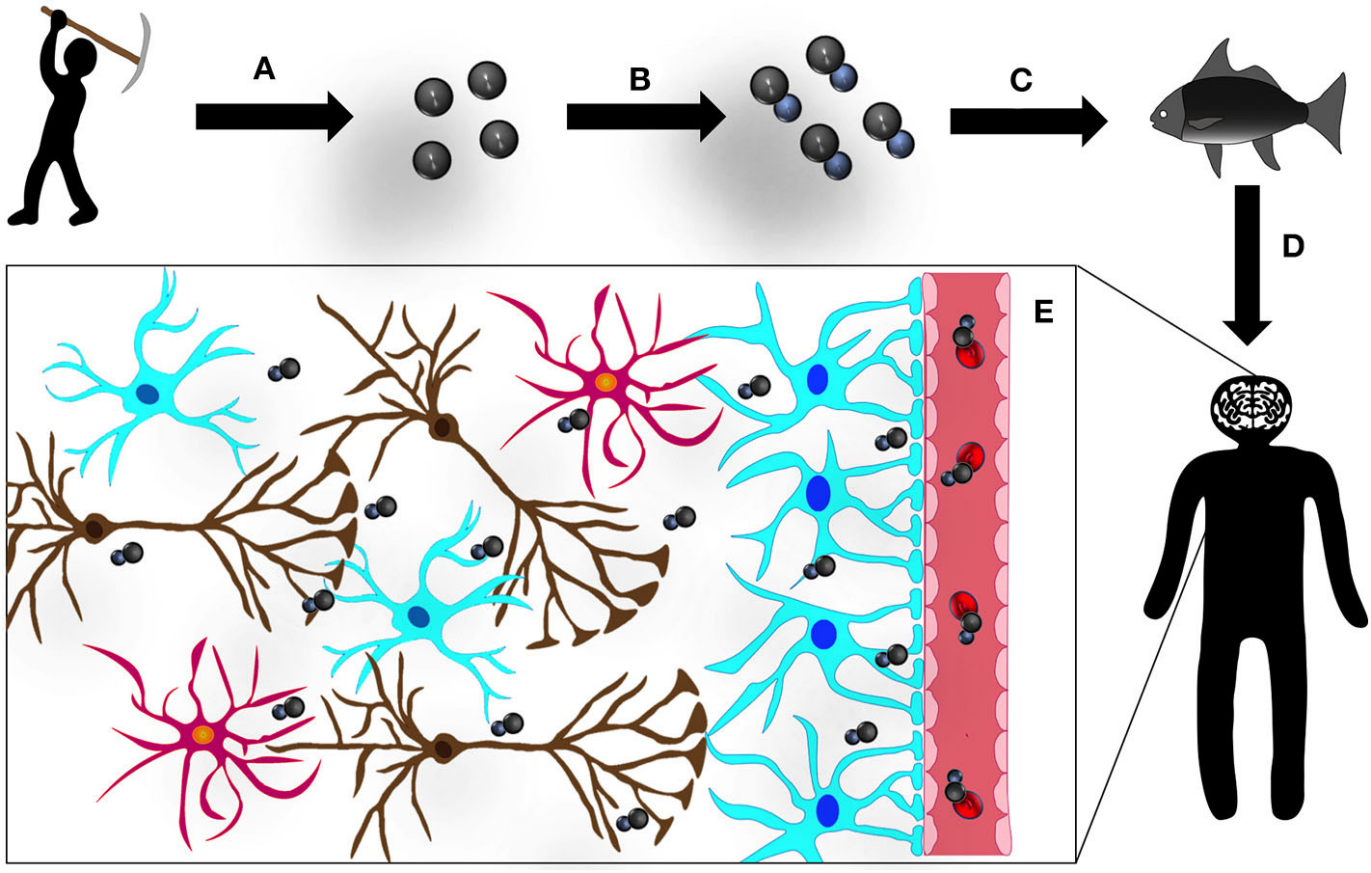
Mercury (Hg): the road to the CNS: **(A)** small-scale and artisanal gold mining releases Hg to the environment, contaminating soil, lakes, and rivers; **(B)** Hg is methylated by bacteria in aquatic sediments converting to its organic form, methylmercury (MeHg); **(C)** this organic MeHg bioaccumulates in predatory fish, undergoing a process of biomagnification and **(D)** enters the human organism *via* contaminated seafood consumption; and **(E)** in the human blood stream, MeHg binds to β-chain hemoglobin in erythrocytes and forms a complex with the aminoacid cystein (complex similar to methionine, MeHg-S-Cys), which can be transported by L-aminoacid transporter (LAT-1) and easily crosses the BBB. Once in the CNS, MeHg disseminates, intoxicating all cell types and structures.

In aquatic environments (lakes, rivers, and oceans) exposed to high levels of MeHg, the food chain, from algae to predatory fishes, bioaccumulates the organic metal over time (Clarkson, 1997). Riverside populations regularly consume predatory fishes (being fish and seafood basic dietary products). Therefore, following ingestion of contaminated food, MeHg is rapidly absorbed by the gastrointestinal tract and rapidly biodistributed in the body, a process further magnified by toxicity-induced vulnerability of the intestinal barrier (Vázquez et al., 2014). Moreover, as we discuss in a recent manuscript, MeHg has been shown to affect microbiota, potentially leading to dysbiosis. This process may impact gut-mediated mercury elimination, through feces, and potentially trigger endotoxins release into the peripheral circulation. This cascade of events may be at the basis of a mounting peripheral inflammatory process and subsequent installation of central chronic neuroinflammation, further contributing to exacerbate MeHg-mediated central neurotoxic processes (Pinto et al., 2020). In addition, MeHg, is highly accumulated in the central nervous system (CNS) due to the special lipid content of the nervous tissue; therefore, MeHg easily reaches the brain due to its ability to cross the blood-brain barrier (BBB), through a L-amino acid transporter (LAT-1) (Kerper et al., 1992; Figure 1) and upregulation of vascular endothelial growth factor (VEGF), reported to cause MeHg-induced weakening of the BBB (Takahashi et al., 2017).

Moreover, the brain, accumulates high levels of MeHg (Lewandowski et al., 2002), leading to strong neurotoxic outcomes (Oliveira et al., 2017). Among the consequences of these neurotoxic effects (even after prenatal exposure to MeHg) one can identify depression-like symptoms (Ceccatelli et al., 2013). Several of these symptoms can be observed in the same individual directly intoxicated with contaminated fish (Foley et al., 2020).

Furthermore, the evidence that chronic exposure of populations to MeHg may lead to early onset of neurodegenerative diseases, such as Parkinson's and Alzheimer's diseases, as well as multiple sclerosis (Carocci et al., 2014; Cariccio et al., 2019), is particularly of great concern.

However, in spite of the solid evidences that MeHg is strongly neurotoxic to neural stem cell (NSC) progenitors, affecting brain development, much less is known about the impact of MeHg in the adult brain. It is particularly relevant to understand how and if MeHg affects the quality of the neurogenic niches, where NSCs and neural progenitor cells (NPCs) persist and produce neurons and glial cells (astrocytes and oligodendrocytes) across the life course. This still highly unexplored research avenue may reveal a strong influence of MeHg in adult neurogenesis, impacting the quality of cognitive resources with putative important consequences for cognitive performance and memory (Sokolowski et al., 2013; Tian et al., 2016).

### Postnatal Brain Neurogenesis

Nowadays, it is well-established that in the adult mammalian brain new neurons are generated continuously in restricted niches and that these newborn cells are functionally integrated into existing neuronal circuits (Gage, 2000). These neurogenic niches are especially active in the subgranular zone (SGZ), located in the dentate gyrus of the hippocampus and in the subventricular zone (SVZ), which is found in the vicinity of the lateral ventricles (Lois and Alvarez-Buylla, 1994; Kempermann et al., 2015).

**Figure 2:**
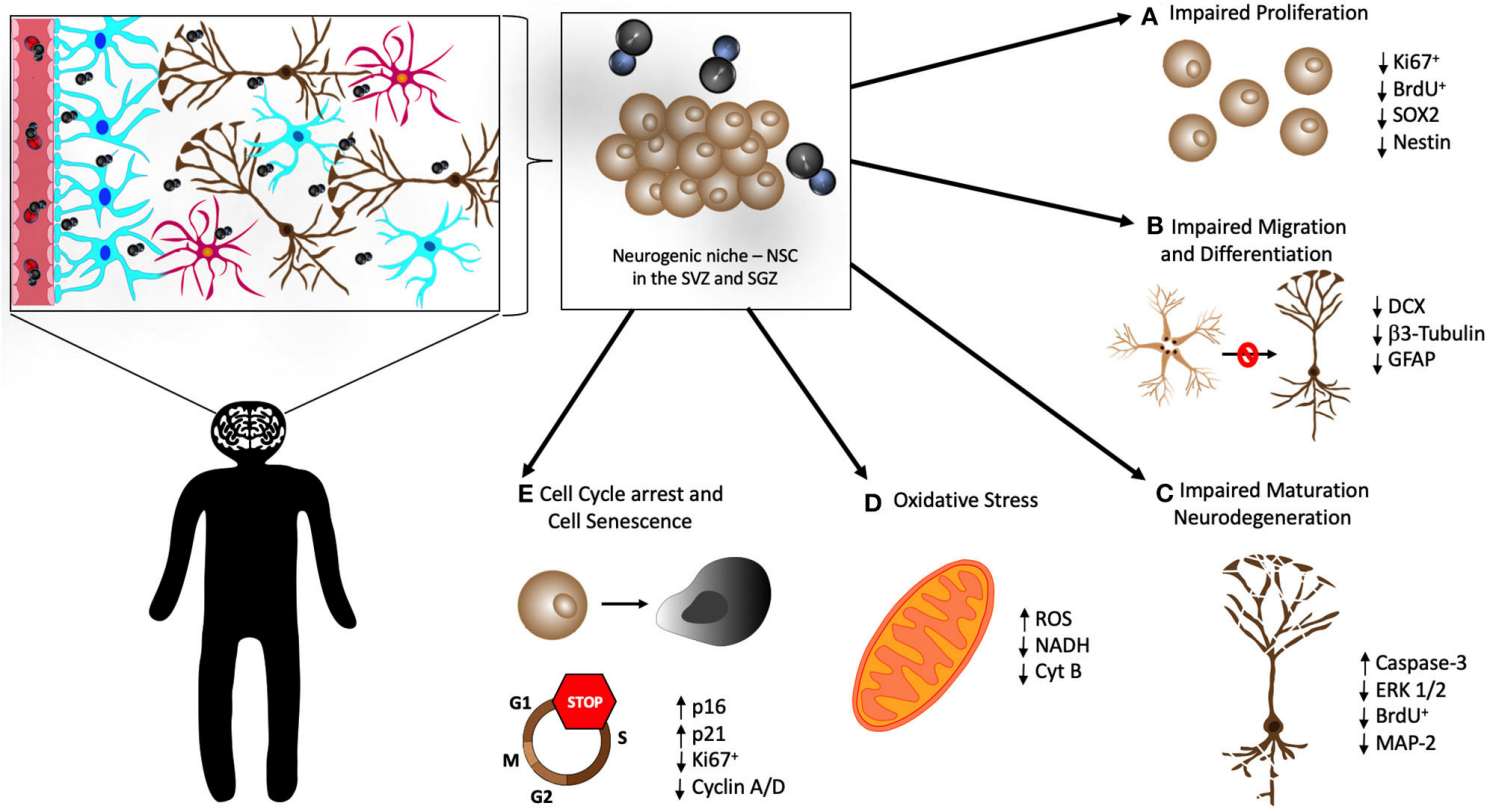
Cellular mechanisms in the neurogenic niche that may be affected by MeHg. Once in the CNS, MeHg reaches the hippocampus and neurogenic niche in the subventricular zone (SVZ) of the lateral ventricles and the subgranular zone (SGZ) of the dentate gyrus. There are several cellular mechanisms that are believed to be altered and impaired by MeHg in neural stem cells (NSC), causing overall poor neurogenesis. There have been observations that MeHg induces **(A)** impaired proliferation of NSC, with a decrease of the proliferation markers Ki67 and BrdU, and of the neural stem markers SOX2 and Nestin; **(B)** impairment of the migration and differentiation of NSC, confirmed by the decrease of the markers DCX, β3-Tubulin, and GFAP; **(C)** disruption of the maturation of new neurons and apoptosis of the existing ones (neurodegeneration), associated with elevated levels of apoptosis related proteins (Caspase-3 and ERK 1/2) and decrease of proliferation (BrdU) and neuronal markers (MAP-2); **(D)** increased oxidative stress, evidenced by elevation of reactive oxygen species (ROS) and decrease in NADH and Cytochrome B (Cyt B); and **(E)** cell cycle arrest, confirmed by the reduction in Ki67 and Cyclin A/D, and cellular senescence of NSC, concomitant with the elevation of the expression of p16 and p21.

Therefore, it is now well-accepted that NSCs persist in the neurogenic niches throughout life and are capable of self-renewal and give origin not only to new neurons but also to glial cells such as astrocytes and oligodendrocytes (Gage, 2002). The activity of the hippocampal neurogenic niche has been extensively studied because this special brain structure plays an important role in modulating higher cognitive functions, notably memory, and affective behaviors (Kempermann et al., 2015) (Figure 2).

In the SVZ, NSCs are the so-called B cells that through intermediate progenitors (C cells) generate neuronal progenitors, the neuroblasts (A cells). These neuroblasts migrate through the rostral migratory stream (RMS) to the olfactory bulb, where they complete their maturation. Therefore, these new cells become functional interneurons and integrate within local neuronal circuits (Doetsch et al., 1999).

In the SGZ, quiescent precursor cells, with radial glial-like cell phenotype, (called type 1 cells) are activated and generate type 2 cells. These cells have high proliferative activity and can generate 2a and 2b phenotypes in their differentiation program, leading to newborn granular neurons and their functional integration into existing hippocampal neuronal circuits (Kempermann et al., 2018).

## Discussion

In spite of the strong academic debate about the relevance of adult hippocampal neurogenesis in humans (Augusto-Oliveira et al., 2019; Moreno-Jiménez et al., 2019), accumulating evidences reinforce its role in human cognition (Luo et al., 2006). Accordingly, it has been reported that the robustness of neurogenesis decreases with aging, eventually leading to impairment in learning, memory, and emotional behavior (Spalding et al., 2013).

Systemic inflammation, often observed with aging (Cevenini et al., 2010), but also in early life in children living in poor sanitation and hygiene conditions (Oriá et al., 2016; McCormick et al., 2019), can mount a neuroinflammatory cascade. This low-grade chronic neuroinflammation impacts the neurogenic niche microenvironment, negatively affecting the formation of new neurons and becoming a chronic modulator of postnatal neurogenesis, as demonstrated in mice models of Alzheimer's disease (Das and Basu, 2008; Valero et al., 2014), but also in older adults exposed pre or perinatally to mercury following Minamata's accident in the 1950s (Yorifuji et al., 2016).

### Methylmercury Targets Brain Neurogenesis? Possible Implications for Accelerated Aging and Cognitive Decline

Primary cultures of rat embryonic NSCs are highly sensitive to MeHg. At micromolar levels, MeHg triggers NSCs apoptosis (Tamm et al., 2006). At submicromolar concentrations, MeHg, although not causing cell death, impacts NSCs dynamics, blunting cell cycle progression. This process involves the inhibition of ERK1/2 phosphorylation, leading to downregulation of cyclin E (Xu et al., 2010).

MeHg also causes a decrease in NSCs proliferation, accompanied with alterations in cell cycle regulators and increase in senescence-associated markers (p16 and p21). Accordingly, MeHg profoundly affects DNA methylation, a process that may induce an epigenetic imprint and disrupts cell programming with potentially long-term consequences for later-life neurodegenerative diseases (Bose et al., 2012).

Altered hippocampal structure and function induced by MeHg intoxication has been extensively reported (Falluel-Morel et al., 2012; Wu et al., 2016; Aragão et al., 2018). For example, a significant reduction in the total hippocampal DNA content has been found in the neonatal rat, 24 h after a single subcutaneous injection of MeHg (5 μg/g) on the postnatal day 7 (Falluel-Morel et al., 2007). Interestingly, this decrease in DNA content was not perceived in the cerebellum and was associated with a significant reduction in Brdu+ cells in the hippocampal dentate gyrus but not in the CA1 and CA3 areas (Falluel-Morel et al., 2007).

Mitochondrial dysfunction has been observed in primary cultures of rat embryonic cortical NSCs exposed to low nanomolar MeHg concentrations, with reduction in NADH dehydrogenase and cytochrome b levels (Bose et al., 2012). Accordingly, oxidative stress and mitochondrial dysfunction in the hippocampus are paramount effects of MeHg toxicity (Vicente et al., 2004; Espitia-Pérez et al., 2018). Interestingly, high levels of MeHg can inhibit STAT3 phosphorylation and increase superoxide production in NSCs *in vitro* (Jebbett et al., 2013). This mitochondrial dysfunction cascade results in activation of caspase-3 and p18, causing apoptosis (Moors et al., 2009).

Mounting evidences strongly associate mercury intoxication with cognitive decline and potential development/progression of dementia, including Alzheimer's disease. The recent paper “One Man's Swordfish Story: The Link between Alzheimer's Disease and Mercury Exposure” reports the interesting case of an old man suffering from cognitive decline with Alzheimer's disease diagnosis showing high levels of mercury due to MeHg-containing fish consumption. Interestingly, following a detoxification dietary regime, his memory partially improved as the levels of mercury dropped in his body (Foley et al., 2020). Accordingly, in another study the authors found a positive correlation between the blood levels of ethylmercury and cognitive decline in older adults and elderly American populations (Geier et al., 2019). Furthermore, other evidences also support a possible long-term effect of mercury toxicity due to pre or perinatal exposure. Accordingly, Yorifuji et al. (2016) reported the occurrence of Minamata older adults with signs of diffuse brain damage and cognitive decline associated with pre or perinatal exposure to MeHg contamination in the 1950s.

All together these findings point to multiple MeHg toxicity mechanisms in brain neurogenesis, suggesting a possible role of MeHg as a strong inhibitor of adult neurogenesis and an inducer of premature cognitive decline with aging under chronic exposure to this toxic heavy metal.

### Conclusions and Future Perspective

Low-grade MeHg chronic intoxication (even being “asymptomatic,” e.g., without clinical neurological symptoms) may be sufficient to disrupt the niches of NSCs in the human brain (especially early in life). Populations exposed to MeHg, including those affected by environmental disasters in Brazil, may be more vulnerable to severe neurological outcomes. Moreover, in addition to the well-recognized neurodevelopmental toxic effect of mercury, it is important to highlight the possible mild cognitive impairments derived from mercury-induced accelerated brain aging. This might be critically relevant for those individuals exposed to the neurotoxicant in important brain development time-windows, due to its impact in compromised neurogenesis and consequent deprivation of the NSC niche in the adulthood.

We still don't fully understand the panoply of effects caused by MeHg in the brain and how these effects may impact adult neurogenesis. These complex effects may also include the potential interaction with lipopolysaccharide and other peripheral gut tract and pathogen-associated inflammatory signals (Li et al., 2019). Novel models to address these issues are needed in future research (Oriá et al., 2018). The use of single cell transcriptomes (Rempel et al., 2015) and optimized *in-vitro* models (Theunissen et al., 2010), including neurospheres derived from NSCs (Moors et al., 2009), may shed light to how microbiota modulates NSCs under MeHg neurotoxicity, including the possible paramount role of microRNAs (Pallocca et al., 2013).

Populations chronically exposed to mercury may suffer limitations in cognitive resources to deal with later aging-related cognitive impairments, and thus exposed to increased risk to develop and cope with neurodegenerative diseases, like Alzheimer's disease. Therefore, this paper calls the attention to the need of increasing awareness for this problem with regulatory authorities in order to closely follow up with great-risk populations, preventing or ameliorating MeHg deleterious effects on brain health.

## Author Contributions

RR: manuscript concept, manuscript writing, and manuscript correction. DP: manuscript writing, manuscript correction, and preparation of figures. RM and RD: manuscript correction and preparation of figures. CF and RO: manuscript correction and funding support. JM: manuscript concept, manuscript writing, manuscript correction, and funding support. All authors contributed to the article and approved the submitted version.

## Conflict of Interest

The authors declare that the research was conducted in the absence of any commercial or financial relationships that could be construed as a potential conflict of interest.

## Publisher's Note

All claims expressed in this article are solely those of the authors and do not necessarily represent those of their affiliated organizations, or those of the publisher, the editors and the reviewers. Any product that may be evaluated in this article, or claim that may be made by its manufacturer, is not guaranteed or endorsed by the publisher.
