# Corrigendum: Methylmercury impact on adult neurogenesis: is the worst yet to come from recent Brazilian environmental disasters?

**DOI:** 10.3389/fnagi.2023.1265755

**Published:** 2023-08-02

**Authors:** Ramon da Silva Raposo, Daniel Vieira Pinto, Ricardo Moreira, Ronaldo Pereira Dias, Carlos Alberto Fontes Ribeiro, Reinaldo Barreto Oriá, João Oliveira Malva

**Affiliations:** ^1^Faculty of Medicine, Center for Innovative Biomedicine and Biotechnology (CIBB) and Institute of Pharmacology and Experimental Therapeutics, Coimbra Institute for Clinical and Biomedical Research (iCBR), University of Coimbra, Coimbra, Portugal; ^2^Experimental Biology Core, Health Sciences Center, University of Fortaleza, Fortaleza, Brazil; ^3^Laboratory of Tissue Healing, Ontogeny and Nutrition, Department of Morphology, School of Medicine, Institute of Biomedicine, Federal University of Ceara, Fortaleza, Brazil

**Keywords:** methylmercury, neurotoxicity, neurogenesis, environmental disaster, memory, aging

In the published article, there was an error in the Funding statement.

“This work was supported by National Funds via FCT (Foundation for Science and Technology) through the Strategic Project UIDB/04539/2020 and UIDP/04539/2020 (CIBB) and Pest UID/NEU/04539/2013; FCT/FUNCAP project POCTI-FEDER-02/SAICT/2017/31699:MercuMemory; POCI-01-0145-FEDER-007440, CENTRO-01-0145-FEDER-0000012:Healthy Aging 2020.”

The correct Funding statement appears below.

